# Small for Gestational Age Newborns in French Guiana: The Importance of Health Insurance for Prevention

**DOI:** 10.3389/ijph.2024.1606423

**Published:** 2024-02-19

**Authors:** Lindsay Osei, Nicolas Vignier, Mathieu Nacher, Juliette Laumonnier, Claude Conan, Loreinzia Clarke, Akoï Koivogui, Sabrina Covis, Luciano Valony, Célia Basurko, Solène Wiedner-Papin, Alain Prual, Thierry Cardoso, Malika Leneuve-Dorilas, Leslie Alcouffe, Najeh Hcini, Stéphanie Bernard, Tiphanie Succo, Françoise Vendittelli, Narcisse Elenga

**Affiliations:** ^1^ Department of Pediatrics, Cayenne Hospital, Cayenne, French Guiana; ^2^ Centre d’Investigation Clinique Antilles Guyane, INSERM, Centre d’Investigation Clinique 1424, Cayenne Hospital, Cayenne, French Guiana; ^3^ Collectivité Territoriale de Guyane, Cayenne, French Guiana; ^4^ Hôpitaux Universitaires Paris Seine-Saint-Denis, Avicenne Hospital, Assistance Publique—Hôpitaux de Paris, Department of Infectious and Tropical Diseases, Bobigny, France; ^5^ Infection, Antimicrobials, Modelling, Evolution, Inserm Unité Mixte de Recherche 1137, Université Sorbonne Paris Nord, Unité de Formation et de Recherche Santé Médecine et Biologie Humaine, Paris, France; ^6^ Caisse Générale de Sécurité Sociale, Cayenne, French Guiana; ^7^ Centre Régional de Coordination des Dépistages des Cancers Ile-de-France, Bondy, France; ^8^ Institut National des Statistiques et Études Économiques, Cayenne, French Guiana; ^9^ Agence Régionale de Santé de Guyane, Cayenne, French Guiana; ^10^ Collectivité Territoriale de Mayotte, Mamoudzou, Mayotte; ^11^ Santé Publique France, Paris, France; ^12^ Réseau Perinat Guyane, Cayenne, French Guiana; ^13^ COREVIH Guyane, Cayenne Hospital, Cayenne, French Guiana; ^14^ Department of Obstetrics, Saint-Laurent-du-Maroni Hospital, Saint-Laurent-du-Maroni, French Guiana; ^15^ Santé Publique France Regional Unit, Cayenne, French Guiana; ^16^ Université Clermont Auvergne, Centre Hospitalier Universitaire Clermont-Ferrand, Centre National de la Recherche Scientifique, SIGMA Clermont, Institut Pascal, Clermont-Ferrand, France; ^17^ Audipog, Université Claude Bernard Lyon 1, Lyon, France

**Keywords:** small for gestational age, newborns, French Guiana, migrants, health insurance

## Abstract

**Objectives:** Small for gestational age (SGA) newborns have a higher risk of poor outcomes. French Guiana (FG) is a territory in South America with poor living conditions. The objectives of this study were to describe risk factors associated with SGA newborns in FG.

**Methods**: We used the birth cohort that compiles data from all pregnancies that ended in FG from 2013 to 2021. We analysed data of newborns born after 22 weeks of gestation and/or weighing more than 500 g and their mothers.

**Results:** 67,962 newborns were included. SGA newborns represented 11.7% of all newborns. Lack of health insurance was associated with SGA newborns (*p* < 0.001) whereas no difference was found between different types of health insurance and the proportion of SGA newborns (*p* = 0.86). Mothers aged less than 20 years (aOR = 1.65 [1.55–1.77]), from Haiti (aOR = 1.24 [1.11–1.39]) or Guyana (aOR = 1.30 [1.01–1.68]) and lack of health insurance (aOR = 1.24 [1.10–1.40]) were associated with SGA newborns.

**Conclusion:** Immigration and precariousness appear to be determinants of SGA newborns in FG. Other studies are needed to refine these results.

## Introduction

Small for gestational age (SGA) newborns are defined as newborns who have a birthweight below the 10th percentile according to their gestational age and sex [1]. Multiple, often interacting risk factors contribute to poor health in women both before and during pregnancy [2]. Contextual factors (root causes) predispose mothers and fetuses to adverse exposure (immediate causes), leading to fetal growth retardation [3]. The contextual factors include broad determinants of health such as political environment, food insecurity, poor water, sanitation and hygiene, environmental pollution, poor maternal education, cultural beliefs, norms and social support given to pregnant women, and access to antenatal care and other health services [3]. Adverse exposures are numerous and are related to maternal nutritional status (such as anemia, low pre-pregnancy body mass-index), maternal infections (such as HIV, *chlamydia*, Trichomonas vaginalis), pregnancy history (young maternal age, primiparity, short inter-birth interval), placenta-related health issues/maternal morbidity (mainly hypertension and pre-eclampsia), uterine and cervical factors (endometriosis and adenomyosis) and toxic exposures such as smoking or alcohol [3, 4]. Fetal factors can also play a role through numerous (epi)genetic abnormalities [1] and multiple pregnancies also play a role [4].

Short-term consequences of SGA include increased risks of hypothermia, polycythemia, and hypoglycemia. A small birth weight increases the risk of later neurodevelopmental problems and cardiometabolic diseases [1] which contributes to the global negative impact on newborns, their families and society at large, resulting in a major loss of human and economic capital [3, 5].

The SGA indicator is mainly used in settings where pregnancy follow-up allows an evaluation of gestational age. This category includes some of the fetuses with fetal growth restriction, a pathologic condition where the fetus is deprived of oxygen and nutrition. However, not all newborns who were growth-restricted fetuses are below the 10th percentile as some non-SGA newborns can be growth-restricted if their intrinsic growth potential was higher. Moreover, approximately 40% of newborns classified as SGA are constitutionally small and healthy [6]. The SGA indicator is more precise than using the WHO low birth weight (LBW) infant definition (newborns with a birthweight below 2,500 g) [7], as the latter also includes some preterm newborns weighing less than 2,500 g but are not SGA [8].

French Guiana is a French overseas territory located in South America in the Guiana shield, bordered by both Brazil and Suriname. Its striking features are an important population growth [9] due to a high fertility rate (27.5 births per 1,000 inhabitants as opposed to a national mean of 10.9 births per 1,000 inhabitants) [10] and the youth of its population, with a median age of 25 as opposed to 40 in mainland France [11]. A significant part of the population migrated from other countries: in 2019, one-third of the population had a foreign nationality and in 2015, one in eight people living in French Guiana was Surinamese, which represents the largest foreign community. The Haitian and Brazilian communities represent 9.3% and 9.2% of the population of French Guiana, respectively [12]. Overall, 53% of the population is reported to live below the French poverty line, as opposed to 14% in mainland France [13]. Also, intense poverty is more likely to strike foreigners as they represent two-thirds of those defined as intensely poor (low income combined with social and material deprivation) [14].

Access to different services, including healthcare, is unequal depending on where people live. For those living on the coast, some may face barriers due to insufficient information or insufficient access to health insurance, or discrimination [15]. Unmet healthcare needs for financial reasons have been reported [16] and contribute to poorer health outcomes with a lower life expectancy [17].

This territory has maternity hospitals distributed in its three major cities (Cayenne, Kourou and Saint-Laurent-du-Maroni) where most women give birth. Cayenne hospital is the only level 3 maternity hospital in French Guiana where are referred newborns requiring neonatal intensive care unit [18]. Women who live in remote cities are admitted to these hospitals prior to delivery to prevent complications, as health facilities located in these remote cities do not have continuous medical presence. However, some births can occasionally occur in these remote health facilities.

In French Guiana, despite substantial efforts to tackle the problem, preterm delivery remains double of that of mainland France [19]. In this context, perinatal causes weigh heavily on premature mortality (before age 65 years) [20]. Infant mortality is 2.6 times higher than in mainland France [20]. Given the frequency of poor pregnancy follow-up, food insecurity and nutritional deficiencies, obstetrical outcomes are still often poor [21]. Although preterm deliveries have been studied in French Guiana, few studies have addressed low birth weight so far. Furthermore, to date, no work has assessed sociodemographic factors associated with low birthweights of children born in this territory which combines widespread poverty and a universal health system. Therefore, the objective of this study was to describe socio-demographic characteristics and health system use associated with SGA newborns in French Guiana.

## Methods

### Materials

The study design is a population based historical cohort study. The studied population included all pregnancies, deliveries and neonatal issues that ended in French Guiana during the collection period.

### Data Source

**FIGURE 1:**
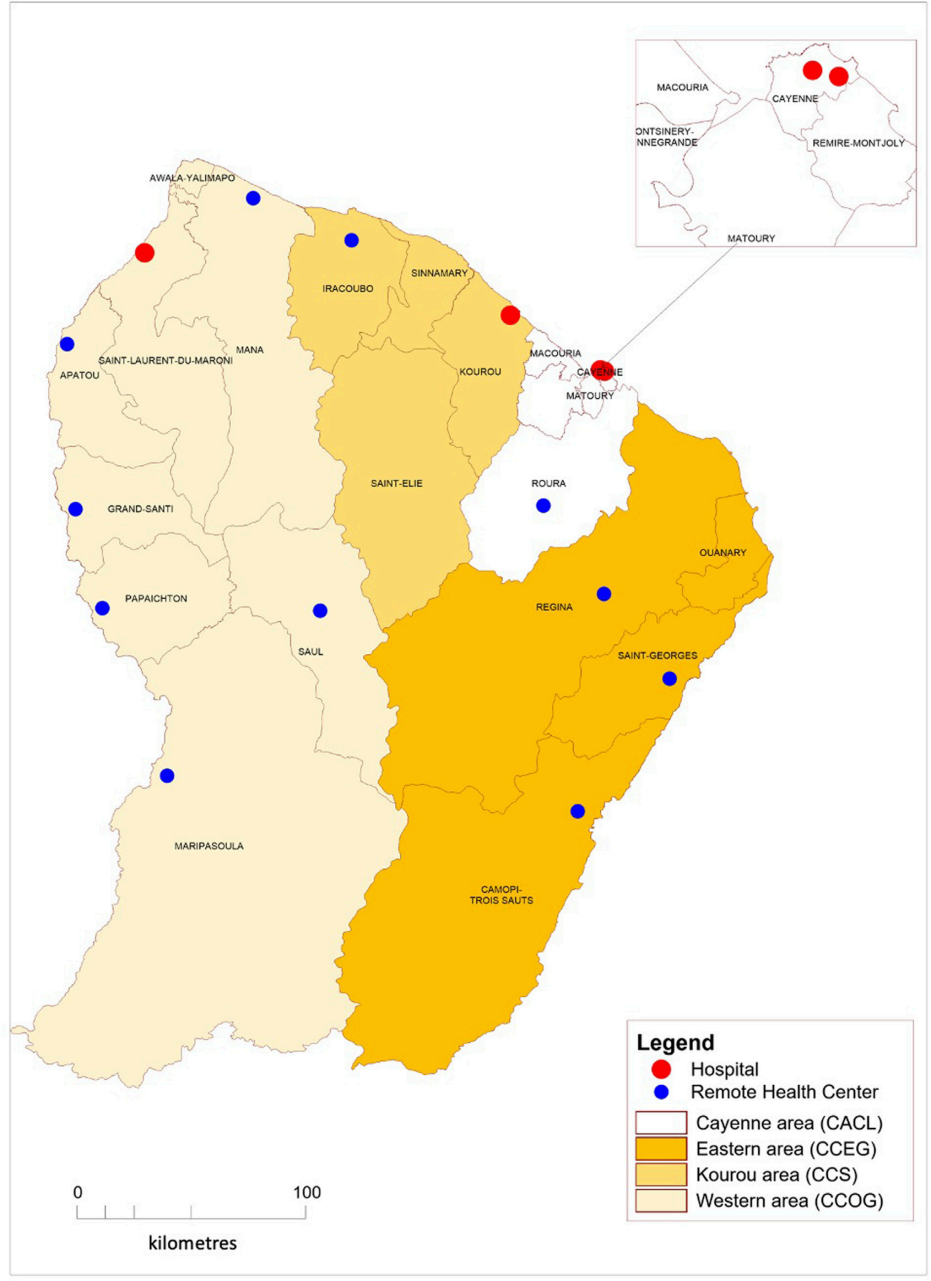
Distribution of health facilities in French Guiana, 2013–2021.

We used data from the complete public territorial birth cohort (Registre d’Issues de Grossesses Informatisé—RIGI) that compiles follow-up data from pregnancies that ended in all maternity hospitals and other health facilities in French Guiana (Figure 1) from 1 January 2013 to 31 December 2021. This database, managed by a local authority (Collectivité Territoriale de Guyane) is filled in by obstetricians or midwives for each birth at the time of delivery. Its objective is to follow-up maternal and neonatal outcomes to improve them.

The variables in the database are the following for each mother: age, place of birth, place of residence (available as four communities of cities matching different local contexts, see Figure 1), health insurance coverage (data only available between 2013 and 2017), health insurance type (state medical assistance for undocumented migrants, universal health insurance coverage for low-income earners and common health insurance) number of prenatal visits, number of performed prenatal ultrasounds and trimester at first medical visit. French guidelines for were used to define cut-off values for pregnancy follow-up items regarding beginning of pregnancy follow-up, number of prenatal visits and number of prenatal ultrasounds [22]. Pregnancies were grouped in two categories: under and over 20 years of age, as defined by WHO to allow international comparisons [23]. For each newborn, the variables are: date of birth, sex, birthweight, gestational age at birth, place of delivery and immediate neonatal outcome (nursery, hospital admission or death in the first 24 h of life).

For each newborn born ≥ 22 weeks of gestation and/or weighing ≥ 500 g (WHO definition [24]), a birthweight percentile was calculated depending on gestational age and sex using an algorithm that has been validated for use in France, including its overseas territories [25]. Two new variables were then generated: small for gestational age (SGA), defined as newborns strictly below the 10th percentile for a given gestational age and sex, and non-SGA, defined as those greater than or equal to the 10th percentile for a given gestational age and sex.

### Methods and Statistics

The primary endpoint was the proportion of SGA newborns.

Qualitative variables were described as percentages and quantitative variables as mean and range. The difference in proportions between the two groups (SGA versus non-SGA) was tested applying Chi-squared tests. Multivariate analyses using logistic regression with maximum likelihood models were used with SGA as dependent variable and socio-demographic data as well characteristics of the pregnancy follow-up as independent variables. Associations were expressed using crude Odds Ratios (cOR), adjusted OR (aOR) and their 95% confidence intervals (95%CI). We computed attributable risk percents to further identify variables with the greatest potential public health interest. When several associated factors explored the same dimension, the choice of a single variable was made. Statistical analyses were performed using STATA^©^ IC 16 software (Statacorp, LLC, College Station, Texas, United States) with statistical significance defined as a *p*-value less than 0·05.

### Ethics

This study was exempt from French Institutional Review approval because our database includes no nominative data, covers all women giving birth in a given time period, and is collected for medical practice assessment. The database has been reported to the French Data Protection Authority (CNIL; Commission Nationale de l’Informatique et des Libertés) as report number 2228099 v 0.

## Results

A total of 67,962 newborns from health facilities throughout French Guiana were entered into the database between 1 January 2013 and 31 December 2021. SGA newborns represented 11.7% of all newborns and mothers under the age of 20 were reported to have a higher proportion of SGA newborns than those over 20 (15.6% vs. 11.1%, *p* < 10^–3^) (Table 1). The proportion of SGA newborns did not significantly differ between preterm birth and those born after 37 weeks of gestation (*p* = 0.73).

**TABLE 1:** Univariate analysis of factors associated with small for gestational age newborns, French Guiana, 2013–2021.

	Total	SGA newborns	Non-SGA newborns	*p*
	N = 67,962	N = 7,925	N = 60,037	
	n	n (%^a^)	n (%^a^)	
Age of mothers	27.8 [12–52]	27.1 [12–49]	27.9 [12–52]	<10^–3^
mean [range]				
Age of mothers	67,962			<10^–3^
<20 years old	8,810	1,377 (15.6)	7,433 (84.4)	
≥20 years old	59,152	6,548 (11.1)	52,604 (88.9)	
Gestation	67,962			0.73
≥37 weeks (term)	59,415	6,938 (11.7)	52,477 (88.3)	
<37 weeks (pretem)	8,547	987 (11.5)	7,560 (88.5)	
Place of delivery	67,962			<10^–3^
Cayenne hospital (level 3)	32,589	4,146 (12.7)	28,443 (87.3)	
St Laurent hospital (level 2)	25,485	2,617 (10.3)	22,868 (89.7)	
Kourou hospital (level 2)	7,345	898 (12.2)	6,447 (87.8)	
St Gabriel hospital (Cayenne - level 1)	2,229	236 (10.6)	1,993 (89.4)	
Remote health centers (level 1)	314	28 (8.9)	286 (91.1)	
Immediate neonatal outcome	67,873			<10^–3^
Nursery	60,412	6,302 (10.5)	54,110 (89.6)	
Hospital admission	5,129	1,388 (21.4)	5,129 (78.6)	
Death	944	229 (25.2)	715 (74.8)	
Place of residence	67,344			<10^–3^
CACL (Cayenne area)	30,889	3,960 (12.8)	26,929 (87.2)	
CCOG (western area)	27,725	2,852 (10.3)	24,873 (89.7)	
CCS (Kourou area)	6,832	861 (12.6)	5,971 (87.4)	
CCEG (eastern area)	1,898	188 (9.9)	1,710 (90.1)	
Mother’s place of birth	66,485			<10^–3^
*France*	30,547			
-French Guiana	26,729	3,005 (11.2)	23,724 (88.8)	
-Antilles	629	60 (9.5)	569 (90.5)	
-Mainland France	3,189	356 (11.2)	2,833 (88.8)	
*Neighbouring foreign countries*	18,466			
Suriname	13,745	1,472 (10.7)	12,273 (89.3)	
Brazil	4,721	405 (8.6)	4,316 (91.4)	
*Non neighbouring foreign countries*	17,472			
Haiti	14,065	2,100 (14.9)	11,965 (85.1)	
Guyana	1,212	182 (15.0)	1,030 (85.0)	
Dominican Republic	1,710	185 (10.8)	1,525 (89.2)	
China	485	25 (5.2)	460 (94.8)	
Other	1,477	135 (9.1)	1,342 (90.9)	
Health insurance coverage	28,838			<10^–3^
No	3,382	435 (12.9)	2,947 (87.1)	
Yes	25,456	2,718 (10.7)	22,738 (89.3)	
Health insurance type	25,456			0.86
State medical assistance	4,093	447 (10.9)	3,646 (89.1)	
(for undocumented migrants)				
Universal health insurance coverage	6,300	668 (10.6)	5,632 (89.4)	
(for low-income earners)				
Common health insurance	15,063	1,603 (10.6)	13,460 (89.4)	
Beginning of pregnancy follow-up	66,383			<10^–3^
First trimester	47,991	5,439 (11.3)	42,552 (88.7)	
Second trimester and beyond	18,392	2,281 (12.4)	16,111 (87.6)	
Number of prenatal visits	59,785			0.05
<7 visits	36,466	4,314 (11.8)	32,152 (88.2)	
≥7 visits	23,319	2,637 (11.3)	20,682 (88.7)	
Number of prenatal ultrasounds	67,025			0.75
<3 ultrasounds	14,480	1,699 (11.7)	12,781 (88.3)	
≥3 ultrasounds	52,545	6,114 (11.6)	46,431 (88.4)	

^a^ Percentages are for each line.

CACL, Communauté d’Agglomération du Centre Littoral; CCOG, Communauté de Communes de l’Ouest Guyanais; CCS, Communauté de Communes des Savanes; CCEG, Communauté de Communes de l’Est Guyanais.

The highest proportions of SGA newborns were observed in 2 maternity hospitals: Cayenne (12.7%), and Kourou (12.2%) and the lowest in remote health centers (8.9%) (*p* < 10^–3^).

As expected, SGA newborns were a large part of newborns admitted to hospital following birth (21.4%), and they also had a higher risk of death (25.2%) when compared to those going to nursery (10.5%) (*p* < 10^–3^).

Newborns from mothers living in the two coastal communities of cities, namely CACL (Cayenne area) and CCS (Kourou area) were significantly more likely to be SGA, with respective proportions of 12.8% and 12.6% (*p* < 10^–3^).

Mothers born in France represented 45.9% of births. The highest proportion of SGA newborns were significantly associated with mothers born in either Haiti (14.9%) or Guyana (15.0%) (*p* < 10^–3^). On the other hand, children born to mothers born in France or neighbouring countries (Suriname or Brazil) had SGA newborns proportions that were all below the proportion observed in French Guiana.

Health insurance coverage data was available for 81.0% of mothers who gave birth between 2013 and 2017: lack of health insurance coverage was significantly associated with SGA newborns when compared to all different levels of health insurance (*p* < 10^–3^). For mothers with health insurance coverage, no difference was found in the proportion of SGA newborns when comparing all three different types of health insurance (*p* = 0.86).

An early pregnancy follow-up (defined as first consultation during the first trimester of pregnancy) was associated with a decreased frequency of SGA newborns (*p* < 10^–3^). However, no association was found between the number of prenatal visits and the number of prenatal ultrasounds and SGA newborns (*p* = 0.052 and 0.75, respectively).

Concerning attributable risk percents for SGA among mothers who had information concerning health insurance coverage, lack of health insurance accounted for 16.8% (*p* < 10^–3^), followed by living in Cayenne area that accounted for 10.7% (*p* < 10^–3^) and a young age (under 20) that accounted for 5.9% (*p* < 10^–3^).

Factors associated with SGA newborns in the multivariate analysis including the health insurance variable (Table 2) were the following: mothers aged less than 20 years old (aOR = 1.65 [95% CI: 1.55–1.77]), mothers born in Haiti (aOR = 1.24 [95% CI: 1.11–1.39]) or Guyana (aOR = 1.30 [95% CI: 1.01–1.68], lack of health insurance coverage (aOR = 1.24 [95% CI: 1.10–1.40] and a late first pregnancy visit (aOR = 1.10 [95% CI: 1.01–1.21]). Conversely, in the same model, other variables were associated with having fewer SGA newborns: mothers who lived in the western area (aOR = 0.78 [95% CI: 0.74–0.82]) or the eastern area (aOR = 0.75 [95% CI: 0.64–0.87]) and mothers born in the Antilles (aOR = 0.83 [95% CI: 0.64–1.09]), Brazil (aOR = 0.74 [95% CI: 0.66–0.83]) or China (aOR = 0.43 [95% CI: 0.29–0.64]).

**TABLE 2:** Multivariate analysis by logistic regression of factors associated with small for gestational age newborns, with (model 1) and without (model 2) the health insurance coverage variable, French Guiana, 2013–2017 and 2013–2021, respectively.

Variable	*N* (%)	cOR [95% CI]	Model 1	Model 2
			aOR [95% CI]	aOR [95% CI]
			*N* = 27,935	*N* = 65,842
Age of mother < 20 years old	67,962			
No	59,152 (87.0%)	Reference	Reference	Reference
Yes	8,810 (13.0%)	**1.49 [1.40–1.59]**	**1.59 [1.43–1.76]**	**1.65 [1.55–1.77]**
Place of residence	67,344			
CACL (Cayenne area)	30,889 (45.9%)	Reference	Reference	Reference
CCOG (western area)	27,725 (87.0%)	**0.78 [0.74–0.82]**	**0.71 [0.64–0.78]**	**0.78 [0.73–0.83]**
CCS (Kourou area)	6,832 (10.1%)	0.98 [0.91–1.06]	1.01 [0.89–1.14]	1.00 [0.93–1.09]
CCEG (eastern area)	1,898 (2.8%)	**0.75 [0.64–0.87]**	1.04 [0.80–1.36]	0.86 [0.73–1.01]
Mother’s place of birth	67,962			
French Guiana	26,729 (39.3%)	Reference	Reference	Reference
Antilles	629 (0.9%)	**0.83 [0.64–1.09]**	**0.65 [0.42–0.99]**	0.85 [0.65–1.12]
Mainland France	3,189 (4.7%)	0.99 [0.88–1.11]	1.00 [0.85–1.18]	1.02 [0.91–1.16]
Suriname	13,745 (20.2%)	0.95 [0.89–1.01]	0.97 [0.87–1.09]	1.06 [0.99–1.14]
Brazil	4,721 (7.0%)	**0.74 [0.66–0.83]**	**0.66 [0.55–0.79]**	**0.74 [0.66–0.83]**
Haiti	14,065 (20.7%)	**1.39 [1.31–1.47]**	**1.24 [1.11–1.39]**	**1.40 [1.31–1.50]**
Guyana	1,212 (1.8%)	**1.40 [1.19–1.64]**	**1.30 [1.01–1.68]**	**1.36 [1.15–1.61]**
Dominican Republic	1,710 (2.5%)	0.96 [0.82–1.12]	0.98 [0.76–1.27]	0.96 [0.82–1.13]
China	485 (0.7%)	**0.43 [0.29–0.64]**	**0.40 [0.23–0.71]**	**0.44 [0.29–0.67]**
Other	1,477 (2.2%)	**0.79 [0.66–0.95]**	0.81 [0.61–1.06]	**0.82 [0.68–0.99]**
Health insurance coverage	28,838			
Yes	25,456 (88.3%)	Reference	Reference	Not included
No	3,382 (11.7%)	**1.23 [1.11–1.37]**	**1.24 [1.10–1.40]**	
Beginning of pregnancy follow up	66,383			
First trimester	47,991 (72.3%)	Reference	Reference	Reference
Second trimester and beyond	18,392 (27.7%)	**1.11 [1.05–1.17]**	**1.10 [1.01–1.21]**	**1.09 [1.02–1.15]**

CACL, Communauté d’Agglomération du Centre Littoral; CCOG, Communauté de Communes de l’Ouest Guyanais; CCS, Communauté de Communes des Savanes; CCEG, Communauté de Communes de l’Est Guyanais.

Bolded values are statistically significant.

## Discussion

Here we showed that, in this French territory with the greatest GDP *per capita* in South America, despite the greatest health expenditure *per capita* and the presence of a universal health system [26], the proportion of SGA was greater than in high income countries (11.7% versus 6.7%), and even greater than South America and the Caribbean (7.8%) [8]. This study also allowed us to document that among some risk factors (mothers aged less than 20, mothers living in Cayenne or Kourou area, late first pregnancy visit) that are associated with having a higher proportion of SGA newborns, three others also stood out as worrisome risk factors: lack of health insurance and place of birth in either Haiti or Guyana.

The latter risk factors, can be combined with the former, as well as with other risk factors associated with SGA newborns such as undernutrition, infections, pre-eclampsia and more [3]. Thus, some parts of the population face an even higher burden, which is all the more worrisome for their children’s outcome.

Foreigners are a large share of the population - one in three inhabitants [12] - in French Guiana, and mostly migrate for economic reasons [27]. Furthermore, they are more likely to live in dire conditions since two-thirds of those defined as intensely poor (low income combined with social and material deprivation) are foreigners [14]. Children born to socially disadvantaged mothers are more likely to face negative outcomes [28], even in high income countries where this has already been demonstrated, with ties to teenage pregnancies [29]. Reports of high teenage pregnancy rates have been made in Haiti [30] and Guyana [31]. Therefore, we can hypothesize that populations coming from non-neighbouring countries have many barriers to integrate the healthcare system, which can be explained by a lack of social support. These very hard living conditions are especially described for women born in either Haiti [32] or Guyana [33], particularly exposed to sexual vulnerability, which can explain the concerning findings for women born in these countries.

Health insurance coverage is normally available for the whole territory of French Guiana, but available data from health insurance services shows a heterogeneous distribution, with a particularly low coverage in the western area of French Guiana [34], mostly due to difficulties to enroll in health insurance coverage (low offer of registration offices) [35] and illegal immigration [36]. Overall, 87% of the population had health insurance coverage in French Guiana, as opposed to 99.9% in France [37]. Here attributable risk percents could suggest that 1 in 6 low birth weight was attributable to lack of health insurance. This difficult access can be explained by a lack of understanding of the administrative process by some parts of the population when submitting their application for health insurance, especially those with low education, resulting in rejection and also with an overload of the system delaying access to health insurance even more [15, 38]. To overcome these barriers, a special access to these services is provided in these isolated areas with agents going directly on site [39].

Moreover, as healthcare can be delivered without prior health insurance coverage in many facilities in French Guiana, some patients, with or without legal status in France, do not actively seek health insurance coverage as they feel their needs are covered with free services, which can be detrimental to them when they need specialised care requiring health insurance coverage [16].

Many reports have highlighted the fact that some groups, especially foreigners, have particularly difficult living conditions, especially in French Guiana where the share of immigrants is as high as almost one-third of the population [12] as opposed to rest of France with only ten percent [14, 40, 41]. French overseas territories have higher proportions of SGA newborns when compared to mainland France [42], we have been able to show that some foreign groups are more affected than others in French Guiana.

A difference in birthweight depending on the place of birth of mothers has already been observed in a high-income country. In a study performed by David et al. in the United States [29], although they did find black women to have newborns with lower birthweights than those born to white mothers, they surprisingly found, even after adjusting for known risk factors, that U.S.-born black women had lower birthweights for their newborns when compared to African-born black women. These data seem to contradict the theory that there is a genetic basis to explain all birthweight differences in a group with similar genetic background as they attribute these differences to different socioeconomic situations. Our results also point to socioeconomic conditions with mothers from Haiti and Guyana having a higher proportion of SGA when compared to the rest in a high-income country such as France [30].

While young age [1] and poor prenatal care [43] have already been associated with SGA newborns, lack of information on certain confounding factors may hamper the significance of our results regarding mothers from Haiti or Guyana. It is likely that mothers born from Haiti may have more pregnancy complications such as pre-eclampsia for instance [44, 45], that could partly explain our results. For mothers born in Guyana, pregnancy-related complications may also explain these results, although literature is very scarce for this population.

A difficult access to health insurance has been shown to be linked to poor neonatal outcomes, even in high income countries such as low birthweight [46], that can also be combined with high infant mortality and premature birth, especially for some migrant populations [47, 48]. An interesting finding from our study is that the proportion of SGA newborns is not different depending on the type of health insurance, which could underline that access to healthcare in itself is an important prevention measure to reduce the incidence of SGA. This finding has also been reported in another high income setting with different levels of health insurance [49].

Other studies did report the association of low-birth weight and SGA newborns with deprived population [50, 51]. Explanations for these associations are numerous, but they mainly revolve around the overlap between the impact of living in a resource-poor settings, the higher prevalence of pregnancy complications and their impact on newborns, and insufficient pregnancy follow-up.

These elements are part of a broader explanation for the reason as to why other perinatal health indicators are far worse in French Guiana when compared to the rest of France [42], given the high proportion of foreign and precarious mothers, especially those born in Haiti, as they gave birth to one-fifth of the newborns during the study. A similar situation of degraded perinatal health indicators is also reported in Mayotte, another French overseas territory located in the Indian Ocean, and the origins are also rooted in the frequent poor socioeconomic conditions [52].

Small vulnerable newborn (SVN) is a new definition and a conceptual framework, bringing preterm birth, SGA, and LBW together. SVN have a markedly increased risk of stillbirth, neonatal death, and later childhood mortality. Additionally these conditions are associated with multiple morbidities with short term and long-term adverse consequences [3].

SVN are numerous in French Guiana, given the high proportion of premature birth of 13% is almost twice as high as the figure found in the rest of France (7%) [19, 53].

Adoption of the framework and the unified definition can facilitate improved problem definition and improved programming for SVN prevention. Interventions aiming at SVN prevention would result in a healthier start for live-born infants. In French Guiana, given the local prevalence of selected conditions also affecting pregnant women (namely syphilis [54], malaria [55], micronutrient deficiency [56] and even hunger [57]) that mostly hit the most disadvantaged, focusing on the following evidence-based antenatal interventions may be useful to reduce the proportion of SVN: syphilis and malaria prevention; micronutriment supplementation as well as balanced diets [58]. The latter could prove very useful in our context as it has been noted in a literature review that maternal nutritional status has the greatest population-attributable fraction for SGA (28.15%), ahead of environmental and other exposures during pregnancy (15.82%), pregnancy history (11.01%), and general health issues or morbidity (10.34%) [4].

Another aspect that will contribute to reducing SVN is ensuring that women have access to health insurance as it can explain some risk factors such as a late first pregnancy visit that is detrimental to a good pregnancy follow-up that can contribute to mitigate the multiple risk factors associated with having SVN with adequate prevention. For instance, pre-eclampsia, is known to be particularly frequent in French Guiana, especially among the most precarious [21], and early interventions have been shown to be essential to limit complications effects associated with this condition [59].

Interventions to reduce the high rate of SVN are currently being implemented: improvement of access to healthcare, as well as prevention against teenage pregnancies with the extension of family planning services to the western part of French Guiana.

### Limitations

The main limitation of our study is the lack of information on other major risk factors published in the literature (such as maternal lifestyle, obstetric factors, placental dysfunction, numerous fetal (epi)genetic abnormalities [1]) for each mother during pregnancy.

There is also a lack of information on some socio-demographic data and migration history. Moreover, the use of attributable risk percents with retrospective studies must be taken with caution and here it was only used to give an intuitive grasp of the magnitude of the problem. Another limitation has to do with comparisons with studies using different growth references in different countries.

### Conclusion

In conclusion, this study showed that SGA newborns are more prevalent among newborns in French Guiana than in other high-income areas or other South America and Caribbean countries. This proportion was highest for mothers aged less than 20 years, mothers living in Cayenne or Kourou area and those with a late first pregnancy visit. It was even higher when three other risk factors were present: lack of health insurance, or place of birth in either Haiti or Guyana. Although prospective studies should aim to refine our knowledge in order to optimize care, the present results emphasize that there is a problem to be tackled. Given these findings, all actors should aim to insist upon the importance of starting pregnancy monitoring as early as the first trimester, and to ensure that all women of childbearing age have access to health insurance. Hopefully, this endeavor will lead to gradual improvements in the health of newborns in French Guiana.
