# Erratum: Small for Gestational Age Newborns in French Guiana: The Importance of Health Insurance for Prevention

**DOI:** 10.3389/ijph.2024.1607279

**Published:** 2024-04-22

**Authors:** Swiss School of Public Health Production Office

**Affiliations:** Frontiers Media SA, Lausanne, Switzerland

**Keywords:** small for gestational age, newborns, French Guiana, migrants, health insurance

Due to a production error, there was a mistake in **Table 1** as published. In the section “‘Mother’s place of birth” in columns “SGA newborns” and “Non-SGA newborns” several rows were incorrectly shifted down. The corrected Table 1 appears below.

**TABLE 1:** Univariate analysis of factors associated with small for gestational age newborns, French Guiana, 2013–2021.

	Total	SGA newborns	Non-SGA newborns	*p*
	N = 67,962	N = 7,925	N = 60,037	
	n	n (%^a^)	n (%^a^)	
Age of mothers	27.8 [12–52]	27.1 [12–49]	27.9 [12–52]	<10^–3^
mean [range]				
Age of mothers	67,962			<10^–3^
<20 years old	8,810	1,377 (15.6)	7,433 (84.4)	
≥20 years old	59,152	6,548 (11.1)	52,604 (88.9)	
Gestation	67,962			0.73
≥37 weeks (term)	59,415	6,938 (11.7)	52,477 (88.3)	
<37 weeks (pretem)	8,547	987 (11.5)	7,560 (88.5)	
Place of delivery	67,962			<10^–3^
Cayenne hospital (level 3)	32,589	4,146 (12.7)	28,443 (87.3)	
St Laurent hospital (level 2)	25,485	2,617 (10.3)	22,868 (89.7)	
Kourou hospital (level 2)	7,345	898 (12.2)	6,447 (87.8)	
St Gabriel hospital (Cayenne - level 1)	2,229	236 (10.6)	1,993 (89.4)	
Remote health centers (level 1)	314	28 (8.9)	286 (91.1)	
Immediate neonatal outcome	67,873			<10^–3^
Nursery	60,412	6,302 (10.5)	54,110 (89.6)	
Hospital admission	5,129	1,388 (21.4)	5,129 (78.6)	
Death	944	229 (25.2)	715 (74.8)	
Place of residence	67,344			<10^–3^
CACL (Cayenne area)	30,889	3,960 (12.8)	26,929 (87.2)	
CCOG (western area)	27,725	2,852 (10.3)	24,873 (89.7)	
CCS (Kourou area)	6,832	861 (12.6)	5,971 (87.4)	
CCEG (eastern area)	1,898	188 (9.9)	1,710 (90.1)	
Mother’s place of birth	66,485			<10^–3^
*France*	30,547			
-French Guiana	26,729	3,005 (11.2)	23,724 (88.8)	
-Antilles	629	60 (9.5)	569 (90.5)	
-Mainland France	3,189	356 (11.2)	2,833 (88.8)	
*Neighbouring foreign countries*	18,466			
Suriname	13,745	1,472 (10.7)	12,273 (89.3)	
Brazil	4,721	405 (8.6)	4,316 (91.4)	
*Non neighbouring foreign countries*	17,472			
Haiti	14,065	2,100 (14.9)	11,965 (85.1)	
Guyana	1,212	182 (15.0)	1,030 (85.0)	
Dominican Republic	1,710	185 (10.8)	1,525 (89.2)	
China	485	25 (5.2)	460 (94.8)	
Other	1,477	135 (9.1)	1,342 (90.9)	
Health insurance coverage	28,838			<10^–3^
No	3,382	435 (12.9)	2,947 (87.1)	
Yes	25,456	2,718 (10.7)	22,738 (89.3)	
Health insurance type	25,456			0.86
State medical assistance	4,093	447 (10.9)	3,646 (89.1)	
(for undocumented migrants)				
Universal health insurance coverage	6,300	668 (10.6)	5,632 (89.4)	
(for low-income earners)				
Common health insurance	15,063	1,603 (10.6)	13,460 (89.4)	
Beginning of pregnancy follow-up	66,383			<10^–3^
First trimester	47,991	5,439 (11.3)	42,552 (88.7)	
Second trimester and beyond	18,392	2,281 (12.4)	16,111 (87.6)	
Number of prenatal visits	59,785			0.05
<7 visits	36,466	4,314 (11.8)	32,152 (88.2)	
≥7 visits	23,319	2,637 (11.3)	20,682 (88.7)	
Number of prenatal ultrasounds	67,025			0.75
<3 ultrasounds	14,480	1,699 (11.7)	12,781 (88.3)	
≥3 ultrasounds	52,545	6,114 (11.6)	46,431 (88.4)	

^a^ Percentages are for each line.

CACL, Communauté d’Agglomération du Centre Littoral; CCOG, Communauté de Communes de l’Ouest Guyanais; CCS, Communauté de Communes des Savanes; CCEG, Communauté de Communes de l’Est Guyanais.

The publisher apologizes for this mistake. The original version of this article has been updated.

